# Cerebrospinal fluid from Alzheimer’s disease patients as an optimal formulation for therapeutic application of mesenchymal stem cells in Alzheimer’s disease

**DOI:** 10.1038/s41598-018-37252-9

**Published:** 2019-01-24

**Authors:** Jeongmin Lee, Soo Jin Kwon, Jang Hoon Kim, Hyemin Jang, Na Kyung Lee, Jung Won Hwang, Jong Hwa Kim, Jong Wook Chang, Duk L. Na

**Affiliations:** 1 Department of Health Sciences and Technology, SAIHST, Sungkyunkwan University, Seoul, Republic of Korea; 2 Department of Neurology, Samsung Medical Center, Sungkyunkwan University School of Medicine, Seoul, Republic of Korea; 3 Neuroscience Center, Samsung Medical Center, Seoul, Republic of Korea; 4 Stem Cell & Regenerative Medicine Institute, Samsung Medical Center, Seoul, Republic of Korea; 5 Department of Obstetrics and Gynecology, Samsung Medical Center, Sungkyunkwan University School of Medicine, Seoul, Republic of Korea; 6 R&D Center, ENCell Co.Ltd, Seoul, Republic of Korea

## Abstract

Mesenchymal stem cells (MSCs) have emerged as one of the promising treatment options for Alzheimer’s disease (AD). Although many studies have investigated on the efficacy of MSCs in AD, how MSCs actually change following exposure to the AD environment has not been studied extensively. In this study, we investigated on the potential of AD patient-cerebrospinal fluid (CSF) samples to be used as a formulation of MSCs and its application in AD therapeutics. When Wharton’s jelly-derived mesenchymal stem cells (WJ-MSCs) were stored in the CSF of AD patients, the stemness of WJ-MSCs was preserved. Furthermore, several genes were upregulated following storage in AD CSF. This signified the therapeutic potential of CSF formulation for AD therapy. Overall, these findings suggest that CSF from AD patients can be an optimal source for MSC formulation.

## Introduction

Originally found in the stroma of bone marrow^1^, mesenchymal stem cells (MSCs) are multipotent progenitor cells which have recently been studied extensively due to their immune modulatory and trophic functions^2^. Alzheimer’s disease (AD) is the most common neurodegenerative disease for which no disease modifying therapy exists so far^3–5^. MSCs have been considered as one of the treatment options for AD since these cells have shown a variety of effects such as reduction of beta amyloid (Aβ) levels, modulation of neuroinflammation, enhancement of endogenous neurogenesis, and also improvement in the behavioral performance of AD-transgenic mice^6–13^.

A growing body of clinical studies is underway to investigate the safety and efficacy of MSCs when administered to AD patients^14–16^. Several preclinical studies have looked into which routes are more feasible to adminster MSCs in AD patients. Animal studies from our group, for instance, showed that intracerebroventricular^17^ or parenchymal injections of MSCs^8^ might be more effective than intravenous^18^ or intra-arterial injections^19^.

While safety, efficacy, and route of administration have been addressed, the clinical grade production of MSCs as a drug product has not been investigated in-depth^20^. From the isolation of cells to the preparation of the final stem cell therapeutics product, a critical point to consider is whether unintended ingredients could be included^21^. Current formulations for MSCs often include supplements to promote the survival of MSCs. However, some supplements may not exist in the body, thus possibilities of undesirable interactions between excipients and internal factors of recipients following injection cannot be ruled out. Therefore, it is imperative to produce an optimized form of the formulation where the excipients have been well estabilished chemically.

When producing clinical grade MSCs, cerebrospinal fluid (CSF) samples from AD patients may have the potential to be applied as an optimal formulation of MSCs for the following reasons. First, one of the most feasible routes of administration for MSCs is the intracerebroventricular route, where cells have higher chances to penetrate into the parenchyma of the brain^22^. To perform repeated intracerebroventricular injections, insertion of a device such as an Ommaya reservoir has to be preceded prior to the first stem cell injection, which makes the collection of CSF from AD patients easier. MSC formulation using the CSF from the patient to whom the MSCs will be injected into may therefore ensure the safety of the therapeutic agent. Second, since CSF flow is part of the Aβ clearance mechanism in AD^23,24^, CSF from AD patients might represent a microenvironment for AD. Therefore, MSCs that have been exposed to the AD patient CSF as a formulation would be preconditioned in the AD microenvironment prior to administration. This pre-exposure of MSCs to the AD microenvironment might allow MSCs to better cope with the disease environment than naïve MSCs.

In the present study, we stored human Wharton’s jelly-derived MSCs (WJ-MSCs) in CSF samples obtained from four different AD patients and three normal controls under hypothermic conditions (4 °C). We then investigated whether the viability and stemness of WJ-MSCs were compromised following exposure to CSF of AD patients or normal controls. In addition, we explored changes in gene expression levels of WJ-MSCs to assess the therapeutic effects of AD CSF formulation.

## Results

### CSF biomarkers of AD patients and normal controls

The four AD patients [male (M): female (F) = 2:2, age range 50–60] who underwent 18F-florbetaben amyloid positron emission tomography (PET) scans were amyloid positive. Negative results were obtained from the three cognitively normal controls (M:F = 2:1, age range 63–81) who also underwent the same amyloid PET scans. Although some samples showed unexpected levels of CSF biomarkers (AD 1, 2: both total and phosphorylated tau lower than AD criteria; Control 1: lower Aβ_42_ level, marked as ^*^ in Table 1), tau/Aβ_42_ ratios (marked as ** in Table 1), which are known to be more accurate than either of tau and Aβ_42_ values, were different between the normal control and AD CSF samples (Table 1). Furthermore, these ratios from all AD patients were above the normal range^25^.

**Table 1:** Demographics of Alzheimer’s disease patients and controls.

	Control 1	Control 2	Control 3	AD 1	AD 2	AD 3	AD 4
Age at sample collections, years	81	63	72	55	60	50	54
MMSE at sample collection	24	29	27	1	4	12	14
Sex	Female	Male	Male	Female	Male	Female	Male
CSF Aβ_42_ level, pg/mL	307.592^*^	844.755	794.12	331.24	370.70	415.54	394.89
CSF t-tau level, pg/mL	107.537	297.624	206.789	285.966^*^	337.525^*^	571.124	533.575
CSF p-tau level, pg/mL	17.75	59.59	37.87	33.13^*^	48.64^*^	84.83	98.97
CSF t-tau/Aβ_42_^**^	0.350	0.352	0.260	0.863	0.911	1.374	1.351
CSF p-tau/Aβ_42_^**^	0.058	0.071	0.048	0.100	0.131	0.204	0.251
CSF cell count and chemistry	Negative	Negative	Negative	Negative	Negative	Negative	Negative
^18^F Florbetaben (Amyloid PET)	Negative	Negative	Negative	Positive	Positive	Positive	Positive

^*^CSF Aβ_42_ and tau levels were within normal limits in normal controls except in Control 1 who showed lower CSF Aβ_42_ according to our norms. On the other hand, AD 1 and 2 showed lower tau levels than expected. However, the ratios of tau to CSF Aβ_42_ (^**^) in all AD samples and controls satisfied the cutoff of AD and normal subjects, respectively.

MMSE, Mini-Mental State Examination; CSF, Cerebrospinal fluid; Aβ_42_, Amyloid beta 1–42; t-tau, total tau; p-tau, phosphorylated tau; ^18^F, Fluorine-18; PET, positron emission tomography.

### Hypothermic storage of WJ-MSCs in AD and normal CSF does not compromise cell viability

**Figure 1:**
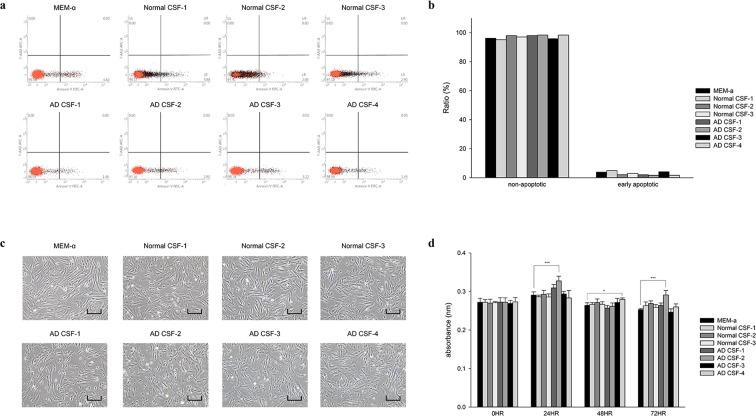
WJ-MSCs stored in AD CSF maintain viability. WJ-MSCs stored under hypothermic conditions (one MEMα 1x media group, four AD CSF samples, and three normal CSF samples) were stained with Annexin V/7-AAD and subsequently analyzed by fluorescence-activated cell-sorting (FACS). Images of the cells were taken, and CCK-8 assays were performed. (**a**) Apoptosis was evaluated after 72 hours. (**b**) Ratio of non-apoptotic/early apoptotic cells from FACS analysis. (**c**) Images of seeded cells were taken (scale bar = 40 μm). (**d**) CCK-8 assay results acquired every 24 hours up to 72 hours. ^*^P < 0.05; ^***^P < 0.001.

Changes in cell viability of WJ-MSCs stored in CSF samples obtained from four different AD patients and three normal controls for 72 hours were assessed by performing fluorescence-activated cell-sorting (FACS) analysis after Annexin V/7-AAD staining (Fig. 1a,b). Images of the cells were taken (Fig. 1c) and the CCK-8 cell counting kit assay was also conducted (Fig. 1d). Compared to WJ-MSCs stored in minimum essential medium alpha 1x (MEMα 1x), significant differences in viability were not observed following hypothermic storage in both AD CSF and normal CSF (Fig. 1). A small percentage of early but not late apoptotic cells was detected from MEMα 1x and CSF groups (both AD and normal) (Fig. 1a,b). Similar results were obtained from the CCK-8 assays, where differences in viability between the MEMα 1x and the two CSF groups were not detected when the assay was performed every 24 hours for up to 72 hours (Fig. 1d).

### WJ-MSCs maintain stemness following hypothermic storage in AD and normal CSF

**Figure 2:**
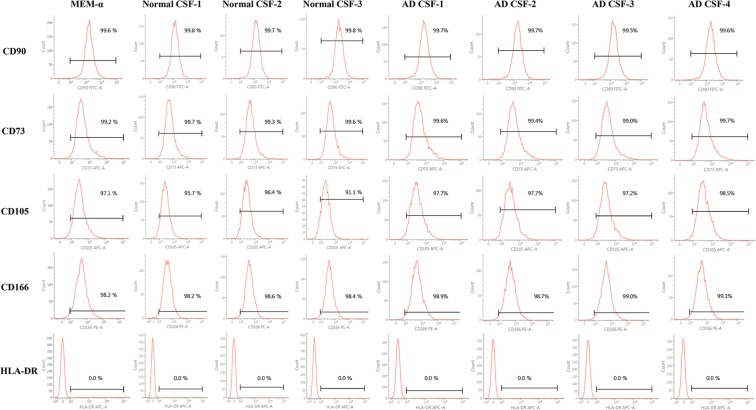
Storage in AD CSF does not alter the cell surface marker expression of WJ-MSCs. Preservation of WJ-MSC stemness was analyzed by using flow cytometry. WJ-MSCs expressed positive cell surface markers (≥90%) and did not express the HLA-DR (0%).

**Figure 3:**
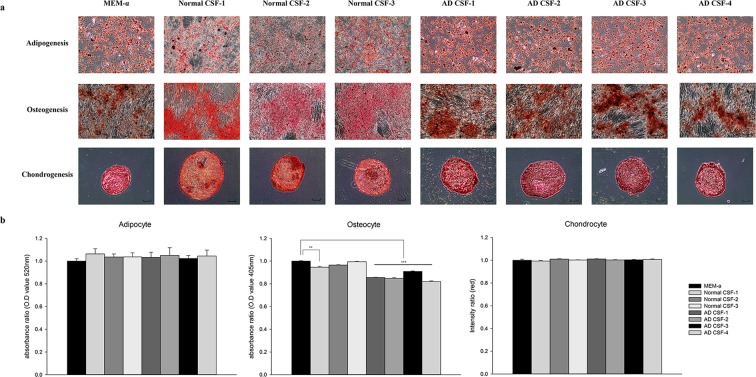
Differentiation potential of WJ-MSCs stored in AD CSF is preserved. Mesenchymal differentiations into adipocyte, osteocyte, and chondrocytes were assessed. (**a**) Differentiations into adipocytes, osteocytes, and chondrocytes, were evaluated by lipid droplet (Oil red O, scale bar = 100 μm), calcium (Alizarin Red S, scale bar = 200 μm), and glucosamine (Safranin O, scale bar = 100 μm) staining methods, respectively. (**b**) Quantitative analysis of the differentiation capacities. One normal CSF sample and AD CSF group showed less differentiated patterns in osteogenesis. ^**^P < 0.01; ^***^P < 0.001.

Immunophenotype characteristics of human WJ-MSCs were analyzed according to the MSC criteria proposed by the International Society for Cell Therapy (ISCT)^26^. Like WJ-MSCs stored in MEMα 1x, WJ-MSCs stored in normal and AD CSF expressed the following cell surface markers: CD90, CD73, CD105, CD166 and also did not express the following hematopoietic markers: CD14, CD11b, HLA-DR, CD34, CD45, and CD19 (Fig. 2 and Supplementary Fig. S1). Such results verified that immunophenotypic features were not altered following exposure to both AD and normal CSF samples. WJ-MSCs were also able to differentiate into various mesenchymal linages (adipogenic, osteogenic, chondrogenic) after exposure to normal and AD CSF samples (Fig. 3). The differentiation efficiency was also similar to that of WJ-MSCs stored in MEMα 1x, although MSCs stored in AD CSF showed less tendency to differentiate into osteocytes.

### mRNA expressions of WJ-MSCs stored in AD CSF were up-regulated compared to WJ-MSCs stored in normal CSF

**Figure 4:**
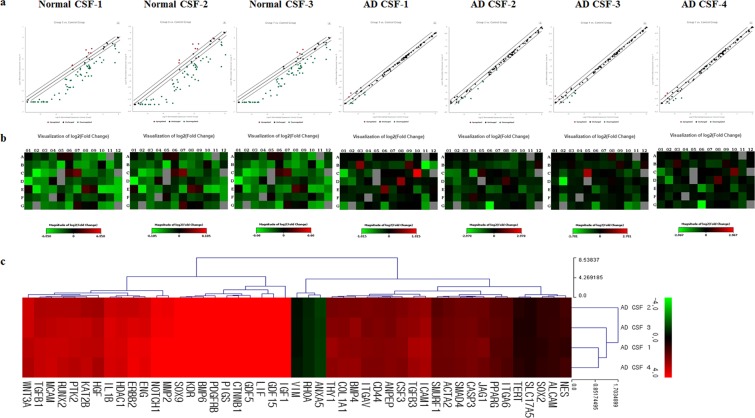
Gene expression patterns of WJ-MSCs stored in AD CSF are upregulated compared to WJ-MSCs stored in normal CSF. Gene expression patterns of WJ-MSCs stored in AD and normal CSF samples were compared to those of WJ-MSCs stored in MEMα 1x. (**a**) Scatter plot and (**b**) heatmap analysis of differentially expressed genes are shown. Overall, while the gene expression levels of normal CSF samples show decreased patterns, a majority of the genes expressed in AD CSF samples remain unchanged. (**c**) Euclidean distance clustering of significant genes performed by MeV software is illustrated as a log transformed data. The green and red colors indicate decrease and increase of gene expression, respectively.

Expression levels of mRNA for MEMα 1x, normal CSF, and AD CSF groups were analyzed by using the Human Mesenchymal Stem Cell RT² Profiler PCR Array. The expression patterns of WJ-MSCs exposed to AD CSF and normal CSF were compared to those of WJ-MSCs exposed to MEMα 1x. Based on the PCR array data, scatter plots and heatmaps were analyzed (Fig. 4a,b). The mRNA expression level of WJ-MSCs exposed to AD CSF was variable among the samples (Fig. 4b). Compared to the WJ-MSCs stored in normal CSF (n = 3), significant alterations in mRNA expression levels were exhibited in 47 genes when WJ-MSCs were exposed to AD CSF (n = 4) (Supplementary Table S1). Interestingly, mRNA expression levels of WJ-MSCs exposed to AD CSF were significantly higher than those of WJ-MSCs exposed to normal CSF. Despite variability of expression levels among the patients’ samples, the pattern of upregulated mRNA was consistent.

### Functional analyses of altered gene expressions show that exposing WJ-MSCs to AD CSF has potential beneficial effects on AD therapy

**Figure 5:**
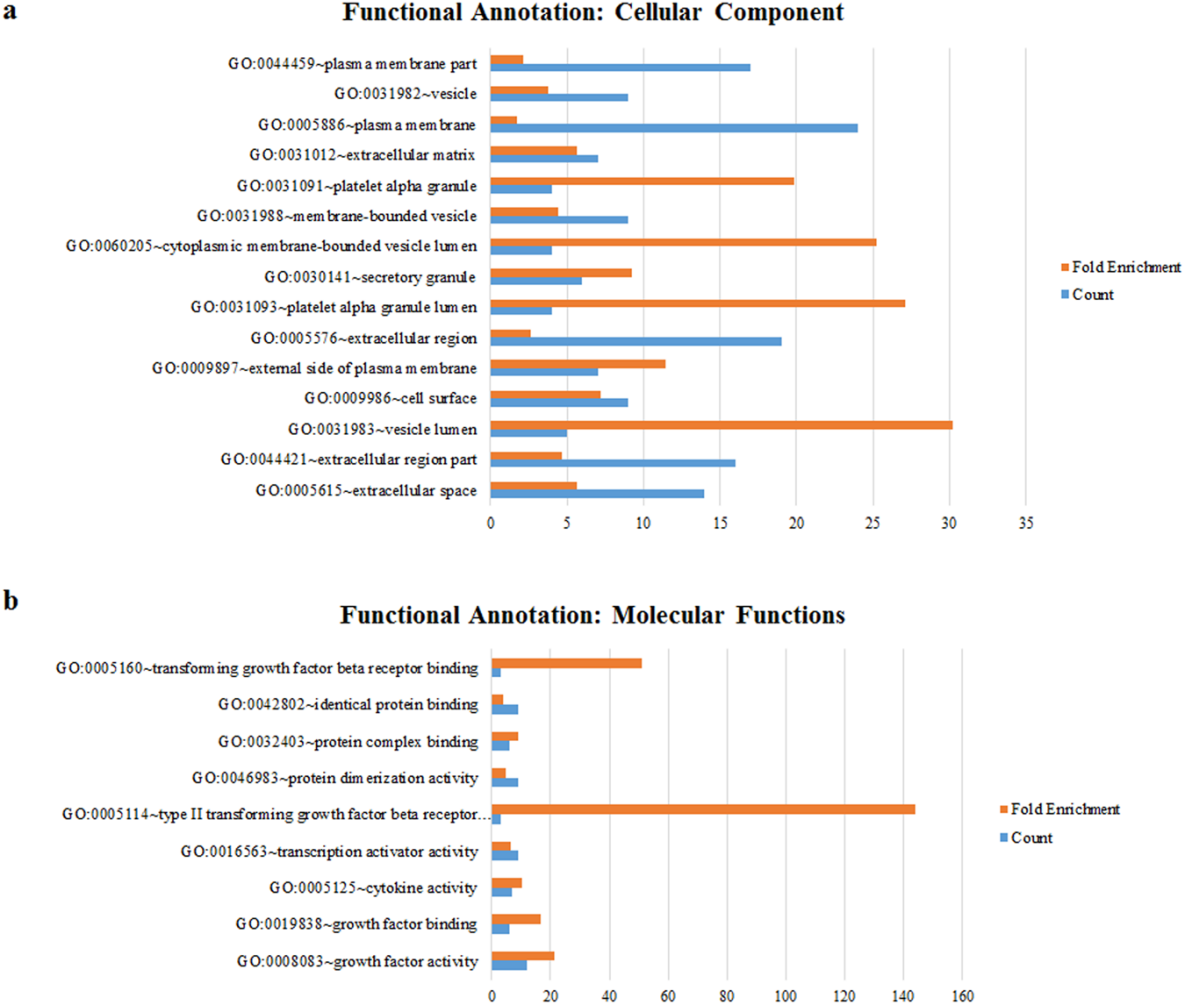
Functional annotation of genes upregulated following storage of WJ-MSCs in AD CSF. Functional analysis performed by using the DAVID informatics tool. (**a**) Cellular component and (**b**) molecular function of up-regulated genes are illustrated in a bar graph.

Gene expression clustering for upregulated and downregulated genes was performed using the MeV software (Fig. 4c). The functions of altered gene expressions were subsequently classified based on cellular component (Fig. 5a), molecular function (Fig. 5b), biological process (Supplementary Table S2) and functional annotation clusters (Table 2).

**Table 2:** Annotation clustering analysis of WJ-MSCs stored in AD CSF.

	Annotation Cluster Summary Term	Enrichment Score
Annotation Cluster 1	Angiogenesis	10.06389447
Annotation Cluster 2	signal peptide	6.479724059
Annotation Cluster 3	negative regulation of programmed cell death	5.554311827
Annotation Cluster 4	regulation of cell proliferation	4.671494633
Annotation Cluster 5	extracellular region	4.589111078
Annotation Cluster 6	tube development	4.17430187
Annotation Cluster 7	morphogenesis of a branching structure	4.167607896
Annotation Cluster 8	cellular component morphogenesis	3.929717283
Annotation Cluster 9	regulation of neurogenesis	3.735311133
Annotation Cluster 10	sensory organ development	3.559638565

Annotation clustering analysis of WJ-MSCs stored in AD CSF using DAVID. The most enriched 10 clusters were shown with summary terms and enrichment scores.

The 10 most enriched annotation clusters of significantly changed genes included items related to angiogenesis, signal peptide, negative regulation of programmed cell death, regulation of cell proliferation, extracellular region, tube development, morphogenesis of branching structure, cellular component morphogenesis, regulation of neurogenesis, and sensory organ development.

## Discussion

Recent studies have reported on the neuroprotective and neurotrophic features of MSCs^27^. Previous studies also showed that proteins secreted from MSCs induced clearance of Aβ proteins^8,9^, promoted neurogenesis and also synaptogenesis^10,13,17^. These studies indicated that a therapeutic interaction existed between the paracrine factors secreted by the MSCs and the endogenous progenitors present in the brain. While former studies were mainly focused on the therapeutic effects of MSCs, the cellular status of MSCs exposed to the AD brain microenvironment has not been fully elucidated.

Several studies have examined the effects of CSF on stem cells. Growth factors found in the CSF have been reported to affect stem cell proliferation^28,29^ and regulate quiescence and activation of stem cells in the brain^30^. Other studies have used CSF to transdifferentiate MSCs into neural cells. For example, MSCs cultured *in-vitro* in CSF (as a substitute of culture media) transdifferentiated into neural-like cells^31,32^. However, these studies have focused on elucidating the effects of CSF on stem cells under normal and not disease conditions.

In this study, we applied CSF samples from AD patients as a formulation of WJ-MSCs. For comparison, MEMα 1x, which is conventionally used for MSC formulation was included, and normal control CSF samples were also included to determine whether AD pathology can possibly alter the therapeutic effects of MSCs. According to the results obtained from this study, the viability and stemness of WJ-MSCs were both preserved after exposure to AD CSF under hypothermic conditions. These results confirmed the safety of AD CSF to be used as a potential source of formulation.

**Figure 6:**
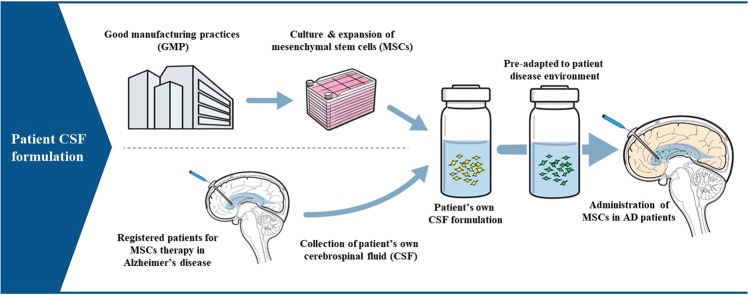
Schematic illustration of AD patient CSF formulation. Using AD patient CSF as a formulation for MSCs allows MSCs to pre-adapt to the patient disease environment.

We further explored changes in gene expression levels of WJ-MSCs exposed to AD and normal CSF samples. Based on analysis of functional annotation clustering, WJ-MSCs stored in AD CSF expressed genes related to enhancement of extracellular transport and signal peptide, which indicates an increase in paracrine activity. These genes are known to exhibit neuroprotective and neurotrophic features such as negative regulation of apoptosis, regulation of cell proliferation, and regulation of neurogenesis. Furthermore, an increase in the expression of genes involved in cell migration or cell adhesion was also observed, which indicates potential beneficial effects on cell survival following administration. These results suggest that AD CSF may act as an optimal formulation for MSCs that will be injected back to the AD patient from whom the CSF sample was obtained from (Fig. 6).

Our study has several limitations. First, our study was based on a small number of patients. Therefore, further research involving a large number of patients is warranted. Second, although we measured the gene expressions of MSCs to investigate functional changes, we have not yet assessed the efficacy of MSCs in animal disease models (e.g., transgenic AD animal models). Nevertheless, to the best of our knowledge, this is the first study which has investigated the fate of MSCs in AD CSF, the interaction between MSCs and CSF samples, and the potential of AD CSF as a formulation source. Our approach may have several advantages. First, patients will be reinfused with their own CSF, which may contribute to minimizing the side effects of allogeneic MSC administration. Autologous MSCs are also available but not as cost-effective due to the requirement of large scale cell expansion, compared to allogenic MSCs. Moreover, the surgical process involved in isolating MSCs is difficult to perform in AD patients due to their age. Second, to achieve repeated injections of MSCs into the lateral ventricle of patients, a device such as an Ommaya reservoir has to be surgically implanted. Therefore, collection of CSF from the patient may not be as invasive as expected. In order to ensure patient safety, CSF collection must be performed under closed, sterile procedures. CSF collected under sterile conditions can be used as an optimal formulation of MSCs produced from the GMP facility. Third, using AD CSF as a formulation of MSCs allows MSCs to pre-adapt to the disease environment and to also become pre-activated prior to administration. The use of CSF from AD patients as a source of formulation may enhance the overall efficacy of AD MSC therapy. This approach can also be applied to a wide range of neurological diseases.

## Methods

### CSF collection from AD patients and evaluation of AD biomarkers

This study was approved by the Institutional Review Board of Samsung Medical Center (IRB approval No. 2015-04-099). In accordance with the guidelines approved by the Institutional Review Board (IRB) of Samsung Medical Center, CSF samples were collected with informed consent from four AD patients and three normal controls. All AD patients fulfilled the National Institute of Neurological and Communicative Disorders and Stroke and the Alzheimer’s Disease and Related Disorders Association (NINCDS-ADRDA) criteria^33^ for probable AD and also had positive 18F-florbetaben PET scans^34,35^. Patients with neurological diseases other than AD were excluded from the study. The three normal controls were recruited from orthopedic clinics in our hospital who underwent spinal anesthesia to receive knee surgeries. All of them met the criteria for normal elderly^36^, had normal Mini-mental State Examination scores defined by age/sex matched cohort^37^, and also had negative 18F-florbetaben PET scans.

A total of 10–12 mL of CSF was obtained from each of the four AD patients and 3 mL from the three normal controls by lumbar puncture (between the L3/L4 or L4/L5 intervertebral space). Within two hours from collection, CSF samples were centrifuged at 4,000 g for 10 minutes at 4 °C. Aβ_1–42_, P-tau_181P_ , and T-tau concentrations were examined from the CSF samples by using the IINOTEST (ELISA) assay. Then the ratios of tau (both t-tau and p-tau) to Aβ_42_ were obtained to validate the difference between normal controls and patients, as these ratios are known to be more accurate than the level of Aβ_42_ and tau^25,38^.

### Hypothermic storage of WJ-MSCs in AD CSF

According to the Institutional Review Board of Samsung Medical Center, after written informed consent was received by all of the patients, umbilical cord tissue was obtained from healthy patients undergoing childbirth. Wharton’s jelly mesenchymal stem cells (WJ-MSCs) were isolated and cultivated according to the standard operation procedures (SOPs) of the Good Manufacture Practice (GMP) facility at Samsung Medical Center.

Passage 5 WJ-MSCs suspended in CSF samples were stored under hypothermic conditions at a concentration of 5.5 × 10^6^ cells/1.5 mL, which corresponded to the final product vial concentration used in the GMP facility. The cells were stored in four different AD CSF samples at 4  C for 72 hours. For the control group, WJ-MSCs were suspended in serum and phenol red free MEMα 1x (Gibco, USA) and three normal CSF samples.

### Evaluation of changes in WJ-MSC viability

Cell proliferation and viability were assessed by using the CCK-8 Cell Counting kit (Dojindo, USA) and Annexin V apoptosis detection kit with 7-AAD (BioLegend, San Diego, CA). MSCs stored for 72 hours under hypothermic conditions from the eight different samples (including the controls) were harvested and analyzed. To determine the number of viable cells in the stocked vials, the cells were stained with Annexin V and 7-AAD antibodies according to the manufacturer’s instructions. The cells were resuspended in 500 μL of 1x staining buffer. Then, 5 μL of Annexin V and 7-AAD antibody were added to the binding buffer for 20–30 minutes at room temperature in the dark, prior to being analyzed by flow cytometry (BD Biosciences, USA).

To analyze the viability of the cells at various time points (0, 24, 48, 72 hrs), MSCs stored in CSF were seeded onto 96-well plates (3 × 10^3^/well), and after 24 hours, CCK8 assay was performed. CCK-8 solution (10 uL) was added to each well, followed by incubation for 1 hour at 37 °C. The absorbance of CCK-8 was measured at 450 nm by using a microplate reader (x-Mark^TM^, Bio-Rad Laboratories, Inc, USA).

### Flow-cytometric analysis for cell surface markers of WJ-MSCs

In order to confirm the stemness of WJ-MSCs, cell surface marker analysis was performed. Harvested MSCs were washed in PBS supplemented with 2% FBS in order to block for non-specific binding sites. Immunophenotypic analysis of MSCs was carried out using flow cytometry for the following markers: CD73, CD90, CD105, CD166, CD14, CD11b, HLA-DR (MHCII), CD34, CD45 and CD19 (BD Biosciences, USA). At least 10,000 events were acquired by using the BD FACS Verse flow cytometer, and the results were analyzed by using the BD FACSuite software version 10. Flow cytometry for appropriate isotype controls were also performed.

### Mesoderm differentiation assays

Another way to test stemness is to examine the differentiation capabilities of MSCs. WJ-MSCs stored under hypothermic conditions for 72 hours were seeded onto 6-well culture plates at a density of 5000 cells/cm^2^ and expanded until cells reached 80–90% confluency. For osteogenic and adipogenic differentiation and the respective immunostaining experiments, cells were incubated in differentiation media according to manufacturer’s instructions (Gibco, USA). Differentiation medium was replaced every 3 days. After 2 weeks, differentiated cells were stained using the following staining methods: osteogenic; Alizarin Red S, adipogenic; Oil Red O. Osteocytes were fixed with 4% paraformaldehyde (PFA) for one hour and then washed with PBS not including both calcium and magnesium (Gibco, USA). Mineralization of the extracellular matrix was visualized by staining with 40 mM Alizarin Red S (Sigma-Aldrich, USA), pH 4.2, for 5 minutes. Adipocytes were fixed with 4% PFA, washed in 60% isopropanol, and subsequently incubated for 10 minutes with Oil-Red O (Sigma-Aldrich, USA) to visualize the lipid droplets. Cells were then washed in isopropanol and counterstained with hematoxylin.

For chondrogenic differentiation, 2 × 10^5^ cells were pelleted in 15 mL conical tubes. Subsequently, the cell pellet were suspended in 500 μL of chondrogenic differentiation medium containing high-glucose Dulbecco Modified Eagle Medium (DMEM; Gibco, USA) supplemented with 500 ng/mL of bone morphogenic protein-6 (BMP-6) (R&D Systems, U.K.), 10 ng/mL of recombinant human transforming growth factor-β3 (R&D Systems, USA), 1% ITS (insulin 25 μg/mL, transferrin 25 μg/mL, and sodium selenite 25 ng/mL), 50 μg/mL of ascorbic acid-2-phosphate, 0.6 μg/mL of dexamethasone, 40 μg/mL of L-proline, and 100 μg/mL of sodium pyruvate. Differentiation medium was replaced every 3 days. Chondrocytes were fixed with 4% PFA, dehydrated by using ethanol, and embedded in OCT compound (Scigen, USA). Blocks were sectioned and the sections were stained with safranin-O (Sigma-Aldrich, USA).

Previously described methods were used to quantify the differentiation potential between each samples^39–41^.

### MSC gene expression analyses

To determine if there is a significant difference in genetic expression of MSCs following hypothermic storage in AD CSF, gene expression analysis was performed. The Human Mesenchymal Stem Cell RT² Profiler PCR Array (PAHS-082, Qiagen, USA) was used to evaluate the expression of 84 specific genes related to MSCs. After 72 hours of hypothermic storage, the total RNA was isolated from 1 × 10^6^ cells by using Trizol (Invitrogen, USA), according to the manufacturer’s instructions. Integrity of isolated RNA was evaluated using the RT2 RNA QC PCR Arrays (Qiagen) and cDNA was synthesized from 500 ng of the total RNA using the RT2 First Strand Kit (Qiagen, USA). The samples were analyzed using the RT2 Profiler PCR Array. The reaction mix was prepared from 2x RT2 qPCR Master Mix and 102 *μ*L of sample cDNA. 10 *μ*L of this mixture was added into each well of the PCR Array. Altogether, 84 different genes were simultaneously amplified in the sample. A melting curve analysis was performed to verify that the product consisted of a single amplicon. PCR arrays were performed in 96-well plates by using a QuantStudio 6 flex real-time PCR system (Applied Biosystems, Thermo Fisher, USA). Data were analyzed via the QuantStudio software and the *Ct* values were determined for each gene. The thresholds and baselines were set according to the manufacturer’s instructions (Qiagen, USA). The fold change in gene expression (compared to the positive control: WJ-MSCs) was calculated using the ΔΔ*Ct* method. Compared to the control (MEMα 1x), gene expressions with a fold change ≥2 was only considered. Clustering analysis of altered gene expressions was performed by using the MeV software (Ver. 4.9.0).

### Functional analyses of altered genes

Functional analysis was conducted to examine alterations in gene expression following storage of MSCs in AD CSF. For annotation analysis, official gene symbols were uploaded into the DAVID (Database for Annotation, Visualization and Integrated Discovery) informatics tool (DAVID Bioinformatics Resources 6.7^42,43^). For GO (Gene Ontology) Term analysis, we studied the Cellular Component (CC), Molecular Function (MF) and Biological Process (BP) categories using the GO FAT default settings. For functional annotation searches, we set the following parameters: CC, threshold count 2, EASE 0.01, Benjamini 0.05 (resulting in 15 chart records); MF, threshold count 2, EASE 0.01, Benjamini 0.05 (resulting in 9 chart records); BP, threshold count 10, EASE 0.01, Benjamini 0.05 (resulting in 60 chart records); for functional annotation clusters, medium stringency (resulting in 52 clusters). Enrichment values (GO Terms), enrichment scores (annotation clusters), and statistical determinants (p values and Benjamini coefficients) were calculated by the DAVID software.

### Statistical analyses

The results are an average of three independent experiments. Data are presented as mean ± standard error of the mean (SEM). Statistical comparisons of each samples between groups were performed using a one-way ANOVA test (both between groups and within groups). Differences were considered statistically significant when P < 0.05. All the statistical analyses were performed using SigmaPlot, version 12.5 and SPSS software, ver 19.0 for Windows.

## Supplementary information

Supplementary Information
