# Supplementary material for: Cerebrospinal fluid from Alzheimer’s disease patients as an optimal formulation for therapeutic application of mesenchymal stem cells in Alzheimer’s disease
Source: Sci Rep. 2019 Jan 24;9:564. doi: 10.1038/s41598-018-37252-9 (PMC6346116; doi:10.1038/s41598-018-37252-9)
Supplement: Supplementary file 1 — Supplementary Information [file 41598_2018_37252_MOESM1_ESM.docx]

**Cerebrospinal fluid from Alzheimer’s disease patients as an optimal formulation for therapeutic application of mesenchymal stem cells in Alzheimer’s disease**

Jeongmin Lee^1,2,3,4^, Soo Jin Kwon^4,6^, Jang Hoon Kim^4^, Hyemin Jang^2,3^, Na Kyung Lee^1,2,3,4^, Jung Won Hwang^1,2,3,4^, Jong Hwa Kim^5^, Jong Wook Chang^1,4,6^*, Duk L. Na^1,2,3,4^*

^1^Department of Health Sciences and Technology, SAIHST, Sungkyunkwan University, Republic of Korea

^2^Department of Neurology, Samsung Medical Center, Seoul, Republic of Korea

^3^Neuroscience Center, Samsung Medical Center, Seoul, Republic of Korea

^4^Stem Cell & Regenerative Medicine Institute, Samsung Medical Center, Seoul, Republic of Korea

^5^Department of Obstetrics and Gynecology, Samsung Medical Center, Seoul, Republic of Korea.

^6^R&D Center, ENCell Co.Ltd, Seoul, Republic of Korea.

Supplementary Information


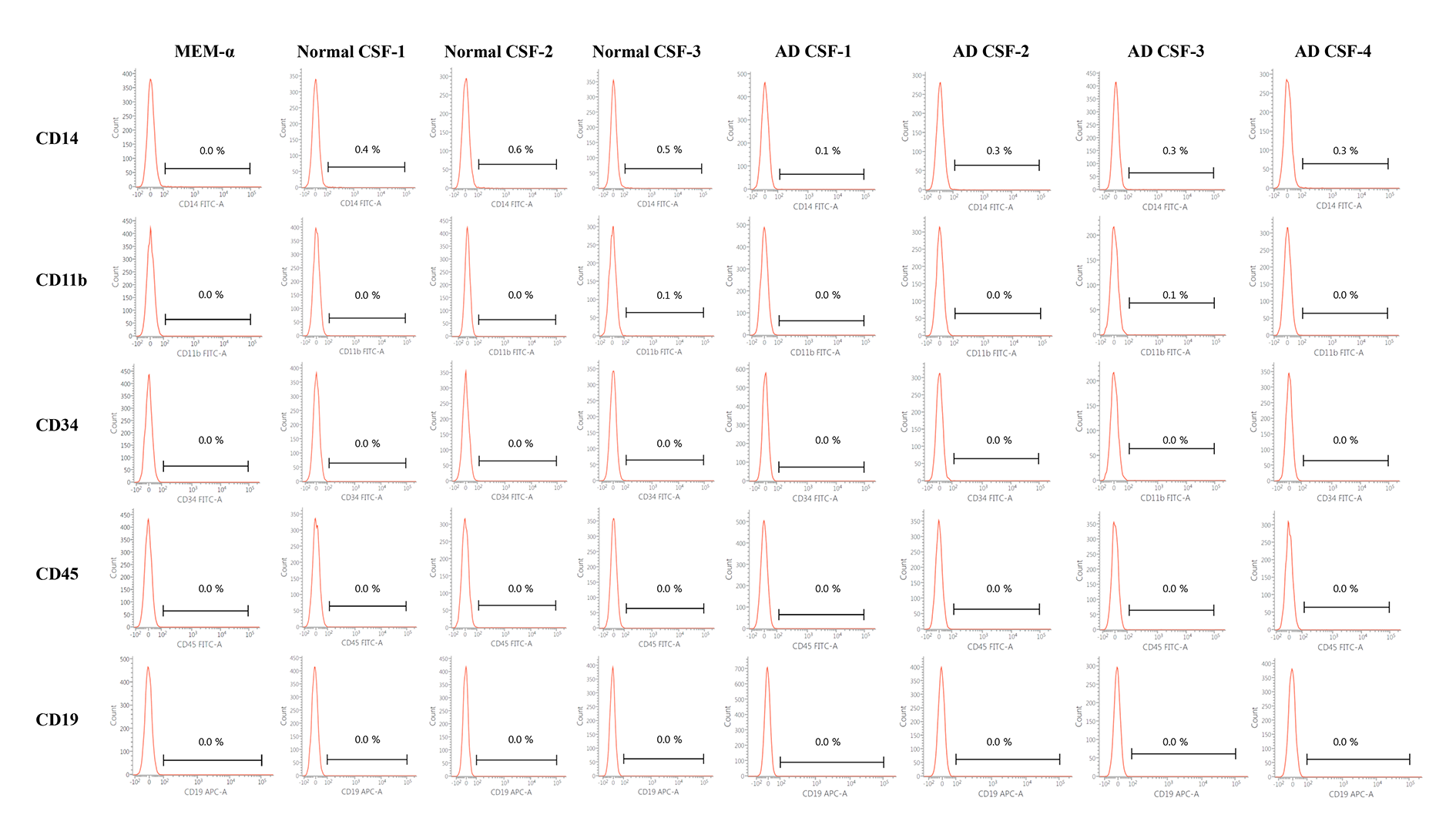


Supplementary Figure S1. WJ-MSCs stored in AD CSF does not express negative markers Negative cell surface markers (CD14, CD11b, CD34, CD45, CD19) of WJ-MSCs are barely expressed after exposure to CSF samples.

**Supplementary Table S1. List of the 47 genes showing significant expression differences between WJ-MSCs exposed to AD CSF and normal CSF**

| **Refseq** | **Symbol** | **Description** | **Fold change** |
| --- | --- | --- | --- |
| NM_001613 | ACTA2 | Actin, alpha 2, smooth muscle, aorta | 3.43 |
| NM_001627 | ALCAM | Activated leukocyte cell adhesion molecule | 1.94 |
| NM_001150 | ANPEP | Alanyl (membrane) aminopeptidase | 4.27 |
| NM_001154 | ANXA5 | Annexin A5 | 0.50 |
| NM_130851 | BMP4 | Bone morphogenetic protein 4 | 4.90 |
| NM_001718 | BMP6 | Bone morphogenetic protein 6 | 24.55 |
| NM_004346 | CASP3 | Caspase 3, apoptosis-related cysteine peptidase | 3.82 |
| NM_000610 | CD44 | CD44 molecule (Indian blood group) | 4.24 |
| NM_000088 | COL1A1 | Collagen, type I, alpha 1 | 4.73 |
| NM_000759 | CSF3 | Colony stimulating factor 3 (granulocyte) | 4.53 |
| NM_001904 | CTNNB1 | Catenin (cadherin-associated protein), beta 1, 88kDa | 32.06 |
| NM_000118 | ENG | Endoglin | 10.99 |
| NM_004448 | ERBB2 | V-erb-b2 erythroblastic leukemia viral oncogene homolog 2, neuro/glioblastoma derived oncogene homolog (avian) | 11.41 |
| NM_004864 | GDF15 | Growth differentiation factor 15 | 42.72 |
| NM_000557 | GDF5 | Growth differentiation factor 5 | 30.04 |
| NM_004964 | HDAC1 | Histone deacetylase 1 | 9.61 |
| NM_000601 | HGF | Hepatocyte growth factor (hepapoietin A; scatter factor) | 6.85 |
| NM_000201 | ICAM1 | Intercellular adhesion molecule 1 | 6.14 |
| NM_000618 | IGF1 | Insulin-like growth factor 1 (somatomedin C) | 85.74 |
| NM_000576 | IL1B | Interleukin 1, beta | 10.19 |
| NM_000210 | ITGA6 | Integrin, alpha 6 | 2.69 |
| NM_002210 | ITGAV | Integrin, alpha V (vitronectin receptor, alpha polypeptide, antigen CD51) | 4.28 |
| NM_000214 | JAG1 | Jagged 1 | 3.62 |
| NM_003884 | KAT2B | K(lysine) acetyltransferase 2B | 7.73 |
| NM_002253 | KDR | Kinase insert domain receptor (a type III receptor tyrosine kinase) | 26.11 |
| NM_002309 | LIF | Leukemia inhibitory factor (cholinergic differentiation factor) | 56.43 |
| NM_006500 | MCAM | Melanoma cell adhesion molecule | 8.29 |
| NM_004530 | MMP2 | Matrix metallopeptidase 2 (gelatinase A, 72kDa gelatinase, 72kDa type IV collagenase) | 14.36 |
| NM_006617 | NES | Nestin | 2.12 |
| NM_017617 | NOTCH1 | Notch 1 | 16.14 |
| NM_002609 | PDGFRB | Platelet-derived growth factor receptor, beta polypeptide | 23.78 |
| NM_033198 | PIGS | Phosphatidylinositol glycan anchor biosynthesis, class S | 34.71 |
| NM_015869 | PPARG | Peroxisome proliferator-activated receptor gamma | 2.88 |
| NM_005607 | PTK2 | PTK2 protein tyrosine kinase 2 | 7.86 |
| NM_001664 | RHOA | Ras homolog gene family, member A | 0.62 |
| NM_004348 | RUNX2 | Runt-related transcription factor 2 | 8.06 |
| NM_012434 | SLC17A5 | Solute carrier family 17 (anion/sugar transporter), member 5 | 1.53 |
| NM_005359 | SMAD4 | SMAD family member 4 | 3.71 |
| NM_020429 | SMURF1 | SMAD specific E3 ubiquitin protein ligase 1 | 3.25 |
| NM_003106 | SOX2 | SRY (sex determining region Y)-box 2 | 1.82 |
| NM_000346 | SOX9 | SRY (sex determining region Y)-box 9 | 26.15 |
| NM_198253 | TERT | Telomerase reverse transcriptase | 1.62 |
| NM_000660 | TGFB1 | Transforming growth factor, beta 1 | 7.84 |
| NM_003239 | TGFB3 | Transforming growth factor, beta 3 | 5.60 |
| NM_006288 | THY1 | Thy-1 cell surface antigen | 4.79 |
| NM_003380 | VIM | Vimentin | 0.79 |
| NM_033131 | WNT3A | Wingless-type MMTV integration site family, member 3A | 8.33 |

**Supplementary Table S2. Functional annotation (“Biological Process”) of WJ-MSCs stored in AD CSF**

| **Term** | **Count** | **P-Value** | **Fold Enrichment** | **Benjamini** |
| --- | --- | --- | --- | --- |
| GO:0045596~negative regulation of cell differentiation | 15 | 7.01E-15 | 19.98818 | 1.07E-11 |
| GO:0042127~regulation of cell proliferation | 22 | 1.53E-14 | 8.046068 | 1.17E-11 |
| GO:0051094~positive regulation of developmental process | 15 | 2.30E-13 | 15.53038 | 1.17E-10 |
| GO:0045597~positive regulation of cell differentiation | 14 | 4.14E-13 | 17.59658 | 1.58E-10 |
| GO:0045165~cell fate commitment | 12 | 8.87E-13 | 24.84861 | 2.71E-10 |
| GO:0001568~blood vessel development | 14 | 9.83E-13 | 16.44742 | 2.50E-10 |
| GO:0001944~vasculature development | 14 | 1.34E-12 | 16.05425 | 2.92E-10 |
| GO:0007167~enzyme linked receptor protein signaling pathway | 15 | 3.86E-12 | 12.62411 | 7.36E-10 |
| GO:0045944~positive regulation of transcription from RNA polymerase II promoter | 15 | 1.15E-11 | 11.63732 | 1.96E-09 |
| GO:0051173~positive regulation of nitrogen compound metabolic process | 17 | 1.51E-10 | 7.597991 | 2.30E-08 |
| GO:0009967~positive regulation of signal transduction | 13 | 1.86E-10 | 12.68402 | 2.58E-08 |
| GO:0045893~positive regulation of transcription, DNA-dependent | 15 | 3.24E-10 | 9.051251 | 4.12E-08 |
| GO:0051254~positive regulation of RNA metabolic process | 15 | 3.61E-10 | 8.975981 | 4.24E-08 |
| GO:0031328~positive regulation of cellular biosynthetic process | 17 | 3.75E-10 | 7.143221 | 4.09E-08 |
| GO:0009891~positive regulation of biosynthetic process | 17 | 4.64E-10 | 7.040441 | 4.72E-08 |
| GO:0010647~positive regulation of cell communication | 13 | 6.50E-10 | 11.37321 | 6.20E-08 |
| GO:0060284~regulation of cell development | 11 | 1.30E-09 | 15.44453 | 1.17E-07 |
| GO:0001525~angiogenesis | 10 | 1.37E-09 | 19.44796 | 1.16E-07 |
| GO:0010557~positive regulation of macromolecule biosynthetic process | 16 | 1.99E-09 | 7.041707 | 1.60E-07 |
| GO:0045941~positive regulation of transcription | 15 | 2.86E-09 | 7.655048 | 2.18E-07 |
| GO:0010628~positive regulation of gene expression | 15 | 4.18E-09 | 7.431062 | 3.04E-07 |
| GO:0006357~regulation of transcription from RNA polymerase II promoter | 16 | 8.38E-09 | 6.334631 | 5.81E-07 |
| GO:0008284~positive regulation of cell proliferation | 13 | 8.73E-09 | 9.038133 | 5.79E-07 |
| GO:0010604~positive regulation of macromolecule metabolic process | 17 | 9.60E-09 | 5.709576 | 6.10E-07 |
| GO:0045935~positive regulation of nucleobase, nucleoside, nucleotide and nucleic acid metabolic process | 15 | 1.04E-08 | 6.918985 | 6.36E-07 |
| GO:0048514~blood vessel morphogenesis | 10 | 3.08E-08 | 13.64122 | 1.81E-06 |
| GO:0042325~regulation of phosphorylation | 13 | 3.25E-08 | 8.029586 | 1.84E-06 |
| GO:0010941~regulation of cell death | 16 | 3.88E-08 | 5.650646 | 2.11E-06 |
| GO:0006928~cell motion | 13 | 4.01E-08 | 7.877447 | 2.11E-06 |
| GO:0035295~tube development | 10 | 4.42E-08 | 13.08317 | 2.25E-06 |
| GO:0051174~regulation of phosphorus metabolic process | 13 | 5.05E-08 | 7.715025 | 2.48E-06 |
| GO:0019220~regulation of phosphate metabolic process | 13 | 5.05E-08 | 7.715025 | 2.48E-06 |
| GO:0001501~skeletal system development | 11 | 8.87E-08 | 9.925165 | 4.23E-06 |
| GO:0007166~cell surface receptor linked signal transduction | 22 | 1.52E-07 | 3.411775 | 7.01E-06 |
| GO:0030182~neuron differentiation | 12 | 1.73E-07 | 7.885748 | 7.78E-06 |
| GO:0043066~negative regulation of apoptosis | 11 | 2.34E-07 | 8.943863 | 1.02E-05 |
| GO:0042981~regulation of apoptosis | 15 | 2.47E-07 | 5.369959 | 1.05E-05 |
| GO:0043069~negative regulation of programmed cell death | 11 | 2.66E-07 | 8.819297 | 1.10E-05 |
| GO:0060548~negative regulation of cell death | 11 | 2.73E-07 | 8.794799 | 1.10E-05 |
| GO:0043067~regulation of programmed cell death | 15 | 2.79E-07 | 5.317053 | 1.09E-05 |
| GO:0031399~regulation of protein modification process | 10 | 5.36E-07 | 9.756942 | 2.04E-05 |
| GO:0000902~cell morphogenesis | 10 | 2.55E-06 | 8.085106 | 9.49E-05 |
| GO:0008285~negative regulation of cell proliferation | 10 | 2.86E-06 | 7.973124 | 1.04E-04 |
| GO:0032268~regulation of cellular protein metabolic process | 11 | 3.33E-06 | 6.679594 | 1.18E-04 |
| GO:0032989~cellular component morphogenesis | 10 | 6.20E-06 | 7.250121 | 2.15E-04 |
| GO:0009611~response to wounding | 11 | 8.93E-06 | 5.973826 | 3.03E-04 |
| GO:0010558~negative regulation of macromolecule biosynthetic process | 11 | 1.18E-05 | 5.788168 | 3.91E-04 |
| GO:0009890~negative regulation of biosynthetic process | 11 | 1.76E-05 | 5.525528 | 5.72E-04 |
| GO:0010033~response to organic substance | 12 | 2.19E-05 | 4.79051 | 6.98E-04 |
| GO:0010605~negative regulation of macromolecule metabolic process | 12 | 2.59E-05 | 4.705664 | 8.07E-04 |
| GO:0010629~negative regulation of gene expression | 10 | 4.14E-05 | 5.710908 | 0.001264 |
| GO:0045934~negative regulation of nucleobase, nucleoside, nucleotide and nucleic acid metabolic process | 10 | 4.68E-05 | 5.621676 | 0.001401 |
| GO:0051172~negative regulation of nitrogen compound metabolic process | 10 | 5.21E-05 | 5.545853 | 0.001526 |
| GO:0031327~negative regulation of cellular biosynthetic process | 10 | 9.48E-05 | 5.130656 | 0.002727 |
| GO:0007155~cell adhesion | 11 | 9.65E-05 | 4.52304 | 0.002723 |
| GO:0022610~biological adhesion | 11 | 9.76E-05 | 4.516587 | 0.002705 |
| GO:0006355~regulation of transcription, DNA-dependent | 17 | 1.45E-04 | 2.759789 | 0.003952 |
| GO:0042592~homeostatic process | 11 | 1.72E-04 | 4.215882 | 0.004603 |
| GO:0051252~regulation of RNA metabolic process | 17 | 1.89E-04 | 2.6989 | 0.00497 |
| GO:0045449~regulation of transcription | 19 | 0.001362 | 2.102563 | 0.034633 |
